# Associations Between Gut Microbiota Composition and Impulse Control Disorders in Parkinson’s Disease

**DOI:** 10.3390/ijms26136146

**Published:** 2025-06-26

**Authors:** Sheng-Hsuan Lin, Ru-Jen Lin, Chia-Ling Chu, Yan-Lin Chen, Shih-Chen Fu

**Affiliations:** 1 Institute of Statistics, National Yang Ming Chiao Tung University, Hsinchu 300093, Taiwan; shenglin@nycu.edu.tw (S.-H.L.); chia.sc12@nycu.edu.tw (C.-L.C.); yan9914@gmail.com (Y.-L.C.); 2 Institute of Data Science and Engineering, National Yang Ming Chiao Tung University, Hsinchu 300093, Taiwan; 3 Department of Applied Mathematics, National Dong Hwa University, Hualien 97401, Taiwan; 4 Department of Biochemistry and Molecular Medicine, National Dong Hwa University, Hualien 97401, Taiwan; 5 Department of Neurology, National Taiwan University Hospital Hsin-Chu Branch, Hsinchu 300195, Taiwan; rujen.lin@gmail.com

**Keywords:** Parkinson’s disease, impulse control disorders, gut microbiota, gut–brain axis, microbiome metabolism

## Abstract

Impulse control disorders (ICDs) are a debilitating non-motor symptom of Parkinson’s disease (PD), often associated with dopaminergic therapy. However, their occurrence in some patients but not others suggests additional biological mechanisms, including the gut microbiome. In this study, we analyzed 191 PD patients (14 with ICDs, 177 without) using 16S rRNA gene sequencing to explore the association between gut microbiota and ICDs. No significant differences were observed in alpha or beta diversity between groups, but several bacterial taxa showed differential abundances. Notably, *Methanobrevibacter* and *Intestinimonas butyriciproducens* were enriched in ICD patients. Functional pathway analysis revealed differences in metabolic pathways, including enrichment of *xenobiotic degradation* and *nicotinate metabolism* in the ICD group. These findings suggest that specific gut microbial taxa and their associated metabolic functions may contribute to ICDs in PD, highlighting a potential non-dopaminergic mechanism and opening new avenues for microbiome-targeted intervention.

## 1. Introduction

Parkinson’s disease (PD) is a neurodegenerative disorder with a sharply rising global prevalence. Between 1990 and 2015, epidemiological studies estimate that the number of PD cases rose more than doubled, reaching approximately 6.2 million worldwide [1,2]. This increase has imposed a significant burden on healthcare systems, not only through direct medical costs but also by adversely affecting the well-being of patients and their families. While PD is primarily characterized by unintended or uncontrollable movements, research also highlights the importance of non-motor symptoms. Many of these non-motor symptoms such as mood disturbances, sleep dysfunction, and cognitive impairment substantially contribute to disease burden. Impulse control disorders (ICDs) have emerged as a particularly disruptive yet often overlooked clinical issue. ICDs in PD manifest as compulsive behaviors that patients struggle to control, regardless of their potentially harmful consequences [3]. Failure to resist a temptation or an impulse often results in pathological gambling, compulsive shopping, binge eating disorder, hypersexuality, and punding, which refers to repetitive, purposeless activities. ICDs can be as prevalent as between 13% and 35% [4,5] among PD patients receiving dopamine agonist.

ICDs are a well-known adverse effect of dopaminergic therapy, particularly dopamine agonists [6,7], which are medications prescribed to alleviate motor symptoms in PD. These compounds activate dopamine receptors in order to compensate patients’ dopamine deficiency. Despite the effectiveness in treating motor symptoms, their influence on the brain’s reward circuitry can also predispose patients to impulsive and compulsive behaviors. As a result, ICD management often involves adjustments to dopaminergic treatment, such as reducing dopamine agonist dosage or transitioning to alternative medications like levodopa. While these strategies may help mitigate impulsive behaviors, they risk exacerbating motor symptoms, underscoring the need for alternative therapeutic approaches. It is also worth mentioning that although dopaminergic therapy triggers ICDs [4,5], this alone does not fully explain why PD patients not receiving dopaminergic treatment still develop ICDs [4,8].

Emerging research suggests that gut microbiota dysbiosis plays a pivotal role in PD pathogenesis and progression, with potential implications for non-motor symptoms [9,10,11,12]. The gut microbiome influences neurotransmitter metabolism and regulation, affecting key molecules such as dopamine, serotonin, and gamma-aminobutyric acid (GABA) [13]. It also modulates brain function through interactions with the hypothalamic–pituitary–adrenal (HPA) axis and neuroinflammation [13]. Furthermore, certain neurotoxic metabolites produced by gut bacteria, including D-lactic acid and ammonia, can enter the central nervous system via the vagus nerve, potentially impairing cognitive and behavioral regulation [14,15,16]. Although substantial evidence links gut–brain axis dysfunction to PD, there is currently no direct evidence connecting gut microbiota dysbiosis to ICDs in PD patients.

Based on these insights, we hypothesize that gut microbiota may regulate impulsive behavior in PD through the gut–brain axis and contribute to ICD development. If validated, this hypothesis would bridge a crucial gap in the current understanding of ICD pathophysiology and support the development of non-pharmacological interventions such as dietary modifications, probiotic supplementation, or other microbiome-targeted therapies. These approaches could provide safer and more sustainable alternatives for managing or even preventing ICDs in PD patients.

Our study aims to investigate the relationship between gut microbiota and ICDs in PD patients. PD patients with and without ICD were recruited, and their gut microbiota composition was analyzed using 16S rRNA sequencing. Functional pathway analysis was also conducted, which allowed us to assess whether gut microbial metabolites are statistically associated with impulsive behaviors. Through conducting the aforementioned analyses, we hope to clarify the role of gut microbiome in managing non-motor symptoms in PD.

## 2. Results

### 2.1. Demography

Of all the 191 PD patients being studied, 14 exhibited impulsive behaviors. Table 1 presents the demographic and dietary characteristics of participants with and without impulsive behaviors. There were no statistically significant differences in gender distribution between the two groups. The proportion of participants aged 65 years or older was also comparable. Antibiotic use was reported by 5.1% of the non-impulsive group, whereas none of the participants in the impulsive group had taken antibiotics. Similarly, there was no significant difference in probiotic use between the two groups. Daily fruit or vegetable consumption, as well as the intake of grains, meat, yogurt, and nuts, showed no significant differences between the two groups. Additionally, there was no significant difference in the use of dopamine agonists or carbidopa-levodopa between the two groups. To minimize potential confounding effects despite the similarities in the aforementioned characteristics between the two groups, sex, age, dopamine agonist use, and carbidopa-levodopa use were included as covariates in subsequent analyses.

### 2.2. Diversity Analysis in Impulsive and Non-Impulsive PD Patients

**Figure 1:**
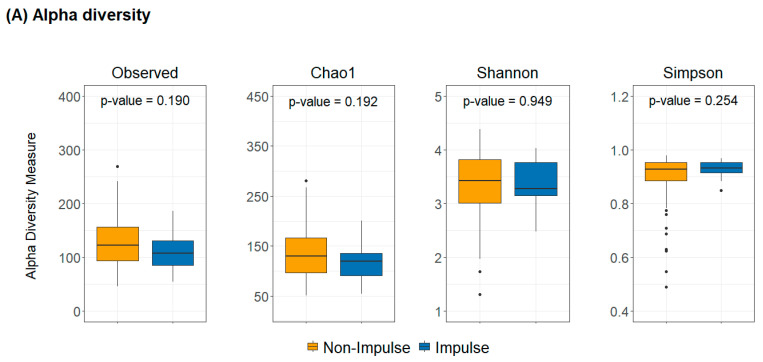

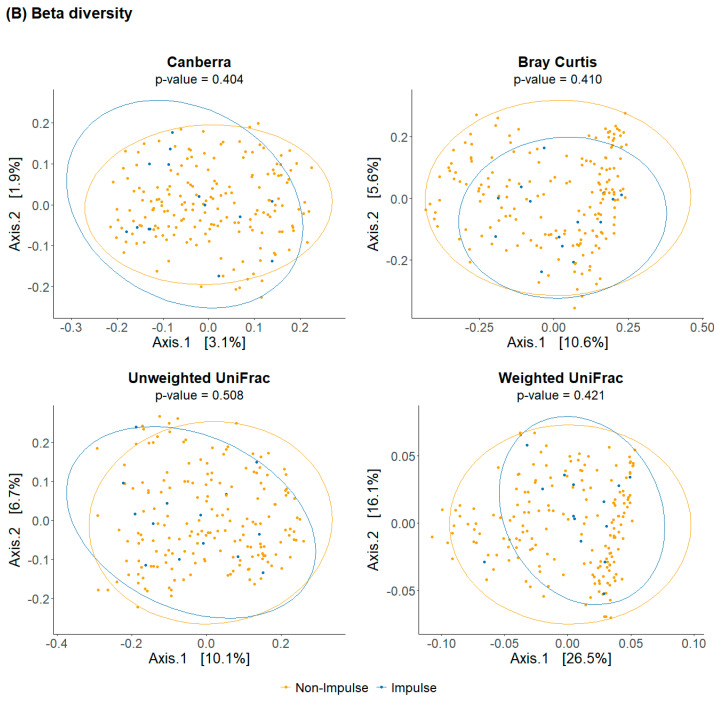
Alpha and beta diversity of gut microbiota in PD patients with and without impulsive behaviors. (**A**) Boxplots of alpha diversity indices (Observed, Chao1, Shannon, and Simpson) show lower diversity in the impulsive group, but the differences were not statistically significant. (**B**) Beta diversity analysis using Canberra, Bray–Curtis, Unweighted UniFrac, and Weighted UniFrac metrics indicates no significant differences between the two groups, suggesting similar overall microbial composition.

Figure 1 presents the alpha and beta diversity of gut microbiota in PD patients with and without impulsive behaviors. Alpha diversity indices, including Observed, Chao1, Shannon, and Simpson, were consistently lower in the impulsive group; however, none of these differences reached statistical significance. These results suggest that impulsive behaviors do not have a substantial impact on gut microbial richness or evenness in PD patients. Beta diversity analysis further confirmed that the overall gut microbial composition did not significantly differ between the two groups. PCoA plots based on Canberra, Bray–Curtis, Unweighted UniFrac, and Weighted UniFrac distances showed no significant clustering differences, indicating that microbial community structures were largely similar between impulsive and non-impulsive PD patients.

### 2.3. Differential Abundance and Network Analysis

**Figure 2:**
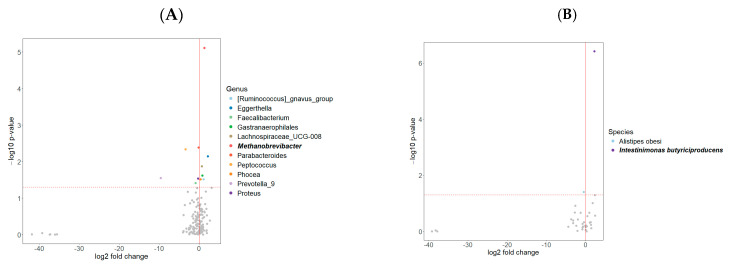
Differential abundance analysis at the genus (**A**) and species (**B**) levels between impulsive and non-impulsive Parkinson’s disease patients, visualized by volcano plots. (**A**) At the genus level, several taxa exhibited significant differences before FDR correction. After correction, *Methanobrevibacter* remained significantly enriched in the impulsive group. (**B**) At the species level, *Intestinimonas butyriciproducens* was the only species that remained significantly enriched in the impulsive group following FDR correction. Genera and species that remained significant after correction are highlighted in bold. In the volcano plots, the horizontal dashed line marks the *p*-value threshold of 0.05, with taxa above this line considered statistically significant. The vertical solid line indicates the log2 fold change of zero. Therefore, taxa on the right are positively associated with impulsivity, while those on the left are negatively associated.

To further investigate gut microbial differences between groups, differential abundance analysis was performed at both the genus and species levels, as shown in Figure 2. Before FDR correction, several genera exhibited significant differences between the impulsive and non-impulsive groups. After FDR correction, *Methanobrevibacter* remained significantly enriched in the impulsive group. Other genera, including *Ruminococcus gnavus group*, *Eggerthella*, *Faecalibacterium*, *Gastranaerophilales*, *Lachnospiraceae UCG-008*, *Parabacteroides*, *Peptococcus*, *Phocea*, *Prevotella 9*, and *Proteus*, also showed differences, though they did not remain significant after correction. At the species level, three species initially showed significant differences before FDR correction. After correction, *Intestinimonas butyriciproducens* was the only species that remained significantly enriched in the impulsive group.

**Figure 3:**
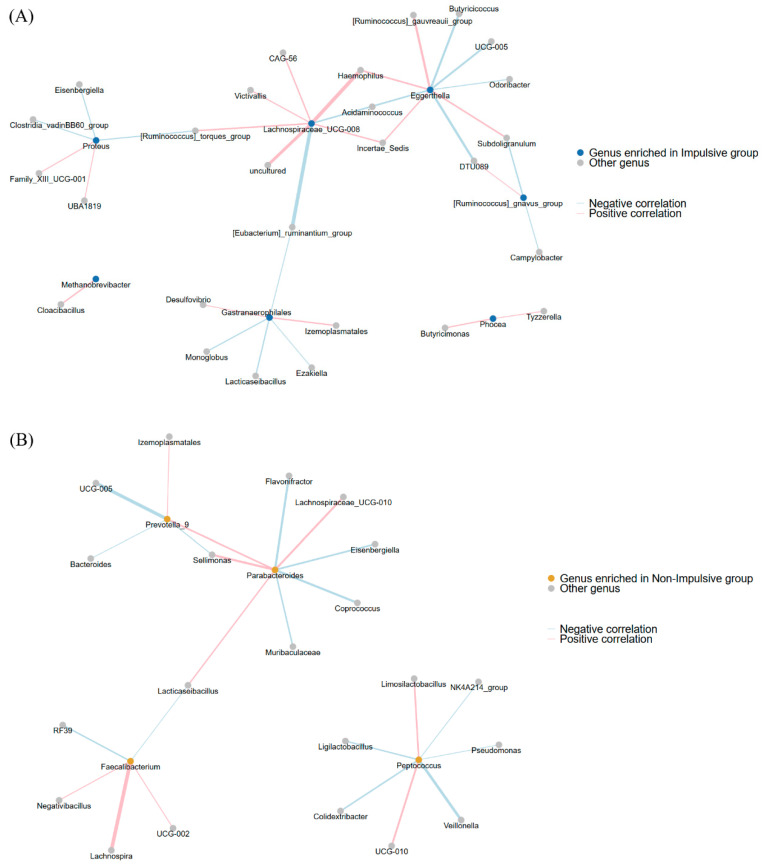
Network analysis illustrating associations gut bacterial genera in (**A**) impulsive group and (**B**) non-impulsive group. Each node represents a genus, with node color indicating whether that genus is enriched in the impulsive group (blue), enriched in the non-impulsive group (orange), or shows no differential abundance (gray). Edges depict Spearman’s rank correlations between genera: pink edges represent positive correlations, and blue edges represent negative correlations; thicker edges indicate stronger associations.

Figure 3A,B show the network analysis of gut bacterial genera that were significantly present in the impulsive and non-impulsive groups, respectively. In the impulsive group, five clusters of genera were identified, with significant correlations among *[Ruminococcus]_gnavus group*, *Eggerthella*, *Proteus*, *Gastranaerophilales*, and *Lachnospiraceae_UCG-008*. *Methanobrevibacter*, which exhibits a significant difference in abundance between the impulse and non-impulse groups after FDR correction, shows a strong positive correlation with *Cloacibacillus*. Together, they form a distinct cluster, separate from other genera. In contrast, the non-impulsive group exhibited three clusters: *Prevotella_9*, *Parabacteroides*, and *Faecalibacterium*. These clusters highlight distinct microbial network patterns between the two groups.

### 2.4. Functional Pathway Analysis

**Figure 4:**
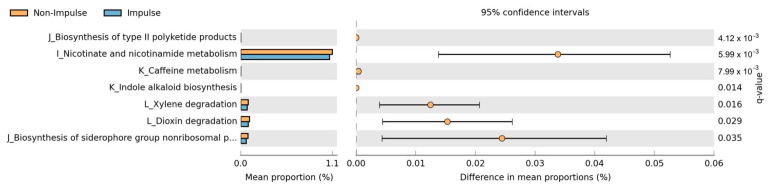
Comparison of metabolic pathway differences between impulsive and non-impulsive Parkinson’s disease patients. Relative abundances of predicted functional pathways were compared between groups using PICRUSt2 and STAMP. After false discovery rate (FDR) correction, six pathways were significantly associated with impulsive behaviors (q < 0.05). The most significant differences were observed in nicotinate and nicotinamide metabolism and biosynthesis of type II polyketide products. The 95% confidence intervals for differences in mean proportions are shown.

Functional pathway analysis using PICRUSt2 and STAMP identified significant differences between the impulsive and non-impulsive groups (Figure 4). After FDR correction, six pathways remained significantly associated with impulsive behaviors. The most significant differences were observed in nicotinate and nicotinamide metabolism and biosynthesis of type II polyketide products, both of which were relatively lower in the impulsive group. Additionally, pathways related to xylene degradation, caffeine metabolism, indole alkaloid biosynthesis, and dioxin degradation were also significantly reduced in the impulsive group.

## 3. Discussion

This study is the first to investigate the association between ICDs and gut microbiota in PD patients. While overall microbial diversity did not differ significantly between impulsive and non-impulsive groups, specific bacterial taxa, including *Methanobrevibacter* and *Intestinimonas butyriciproducens*, were significantly enriched in impulsive patients. Functional pathway analysis further revealed that impulsivity was linked to alterations in microbial metabolic functions related to nicotinate and nicotinamide metabolism, polyketide biosynthesis, and xenobiotic degradation. These findings suggest that gut microbiota may be involved in ICD in PD through taxonomic shifts and metabolic dysregulation. Given the established role of the gut–brain axis in PD pathophysiology, the observed microbial differences could potentially influence neurotransmitter metabolism, neuroinflammation, or other pathways implicated in impulsivity.

Our findings suggest that taxonomic differences rather than overall microbial diversity may play a role in impulsivity among PD patients. This is consistent with the results from the study of imprisoned impulsive versus non-impulsive women [17], in which no difference in alpha nor beta diversities were found between the groups. The apparent lack of diversity differences alongside taxonomic shifts is not contradictory and is in fact a commonly reported pattern in microbiome studies. Diversity metrics reflect the overall richness and evenness of microbial communities, but they may not capture specific compositional changes in individual taxa that could be biologically meaningful. Thus, the identification of distinct taxa such as *Methanobrevibacter* and *Intestinimonas butyriciproducens* in the absence of global diversity changes is consistent with prior findings and supports the notion that targeted microbial shifts, rather than broad diversity alterations, may underlie behavioral phenotypes such as impulsivity. Interestingly, the same research group reported that *Methanobrevibacter* abundance was positively correlated with isobutyric and isovaleric acids—branched chain fatty acids that may contribute to colonic epithelial stress under certain conditions [18].

Given the emerging evidence linking gut microbiota to neurobehavioral regulation, it is plausible that these microbial alterations contribute to impulsivity through their metabolic byproducts and interactions with the gut–brain axis. Short-chain fatty acids (SCFAs), particularly butyrate, have been shown to influence brain function and behavior by modulating neuroinflammation, blood–brain barrier integrity, and neurotransmitter production [19]. Dysbiosis of SCFA-producing bacteria may lead to abnormal SCFA production, which in turn could affect neural signaling and impulse control. Our results showed that *Intestinimonas butyriciproducens*, a butyrate-producing bacterium [20], was significantly enriched in the impulsive group. Butyrate has been implicated in regulating GABA and serotonin levels, both of which play crucial roles in impulse control and emotional regulation [21]. The altered abundance of *Intestinimonas butyriciproducens* and other SCFA-related bacteria in our study suggests a potential link between gut microbial metabolism and impulsive behaviors in PD.

The functional pathway analysis identified significant metabolic differences between impulsive and non-impulsive PD patients, particularly in nicotinate and nicotinamide metabolism, polyketide biosynthesis, caffeine metabolism, and indole alkaloid biosynthesis. These pathways are involved in neurotransmitter regulation, antioxidant defense, and detoxification, suggesting that microbial metabolism may influence behavioral control mechanisms. Notably, caffeine metabolism was significantly reduced in the impulsive group, which may indicate alterations in adenosine receptor signaling. Caffeine has been reported to modulate dopamine release [22,23], both of which are linked to impulse control. Given that gut microbiota influence caffeine metabolism, these findings raise the possibility that microbial changes in PD could indirectly affect neurochemical pathways involved in impulsivity. However, it remains unclear whether these differences are a cause or consequence of impulsive behaviors in PD.

Alterations in indole alkaloid biosynthesis suggest a potential link between gut microbial tryptophan metabolism and serotonin regulation. The gut microbiota contribute to the production of indole-3-acetic acid (IAA), indole-3-propionic acid (IPA), and indole acetaldehyde (IAAld), which are implicated in neuroprotection, oxidative stress reduction, and serotonin synthesis [24,25]. Since serotonin plays a key role in mood and impulse regulation, disruptions in microbial-derived indole metabolism could influence ICD symptoms in PD patients. Prior studies have shown that serotonin production is modulated by specific gut bacteria, and dietary tryptophan intake can impact motor and behavioral outcomes in PD models [24,25]. The observed reductions in this pathway in the impulsive group suggest a possible microbial contribution to serotonin dysregulation, though further research is needed to determine its clinical relevance and potential therapeutic implications.

Our findings further contribute to the emerging literature connecting gut microbiota to behavioral regulation. A recent study by Konstanti et al. (2024) [26] investigated impulsivity in older adults with metabolic syndrome and reported significant beta diversity differences associated with cognitive performance, along with specific bacterial genera (e.g., *Butyricicoccus*, *Blautia*) and functional pathways (e.g., glucuronate and galacturonate metabolism) linked to impulsive traits. While their study demonstrated both broad compositional and targeted taxonomic shifts, our results did not show significant diversity differences but instead emphasized specific microbial taxa and functional changes associated with impulsivity in PD. Together, these complementary findings suggest that microbial signatures of impulsivity may vary depending on the population and phenotype assessed. In line with this, a recent systematic review by Langmajerová, Roubalová, Šebela, and Vevera (2023) [27] highlighted the limited but growing evidence on the relationship between gut microbiota and impulsive or violent behavior, particularly in neuropsychiatric conditions. Our study extends this research frontier by characterizing microbiome-behavior associations in Parkinson’s disease—a context not previously explored—thereby contributing to a broader understanding of how the gut–brain axis may influence behavioral regulation across diverse clinical populations.

Although this study provides insights into the association between gut microbiota and impulsive behaviors in PD patients, several limitations should be considered. First, the sample size was relatively small, which may have limited the statistical power to detect subtle microbiome-behavior associations. Second, the cross-sectional design precludes the establishment of causality. It remains unclear whether gut microbial alterations contribute to the development of impulsivity or arise as a secondary effect of behavioral or physiological changes in PD. Longitudinal studies are necessary to assess temporal changes in microbial composition and metabolic pathways in relation to impulse control disorders. Additionally, metabolic pathway predictions were derived from bioinformatics tools rather than direct metabolite quantification. While predictive approaches provide valuable functional insights, they do not capture dynamic metabolic processes or confirm biochemical activity in vivo. Future studies should incorporate targeted metabolomics to validate microbial contributions to neurotransmitter metabolism and behavioral regulation. Furthermore, potential confounding factors such as dietary habits, medication use, and disease severity were not fully accounted for. In particular, dietary intake data were obtained from a self-reported food frequency questionnaire that captured whether participants consumed certain food groups (e.g., fruits, grains, meats) on a daily basis. While this provides some insight into general dietary habits, the data lacked detail on portion sizes, preparation methods, or total nutrient intake. These limitations constrained our ability to fully adjust for dietary influences on the gut microbiota. Addressing these limitations in future research will be crucial to understanding the complex interplay between the gut microbiome and impulsivity in PD.

The findings of this study indicate that specific gut microbial taxa and metabolic functions may be associated with impulsivity in PD, despite the fact that overall microbiome diversity was not significantly different between groups. This pilot study sheds light on the possibility of future microbiome-targeted interventions, such as dietary modifications or probiotics, on influencing impulsivity in PD patients.

## 4. Materials and Methods

### 4.1. Participant Recruitment and Data Collection

We adapted data from the study conducted by Hill-Burns et al. [28], which initially involved 376 participants recruited through the NeuroGenetics Research Consortium at multiple sites in the United States, including Seattle, Washington; Atlanta, Georgia; and Albany, New York, between March 2014 and January 2015. A total of 45 individuals were excluded: 41 due to having fewer than 5000 sequencing reads and 4 Parkinson’s disease (PD) patients with missing data. The methodologies, along with the clinical and genetic characteristics of the NeuroGenetics Research Consortium dataset, have been comprehensively documented by Hamza et al. [29]. After these exclusions, the final dataset included 331 participants, consisting of 200 individuals diagnosed with PD based on the modified UK Brain Bank criteria [30] (134 males, 66 females; mean age: 68.35 years) and 131 self-reported healthy individuals without neurodegenerative conditions (52 males, 79 females; mean age: 70.37 years). Since none of the healthy controls exhibited impulsive behaviors, further analyses were limited to the 200 PD patients. Impulse symptoms were evaluated using the Gut Microbiome Questionnaire, and an additional 9 participants were excluded due to missing impulse status data. Consequently, the final dataset consisted of 191 PD patients (128 males, 63 females; mean age: 68.04 years). Dietary intake was assessed using a self-reported food frequency questionnaire. Participants reported the frequency of consumption across major food groups (e.g., grains, nuts, meats, fruits, and vegetables). Detailed information on portion sizes or nutrient intake was not available. Further details regarding fecal sample collection, DNA extraction, sequencing, and metadata collection can be found in Hill-Burns et al. [28]. To sum up, the exclusion process is as follows:

- Excluded 41 participants with <5000 sequencing reads
- Excluded 4 PD patients with missing clinical data
- Excluded 131 healthy controls (not included in final analysis)
- Excluded 9 PD patients with missing impulse behavior data
- Final analysis included 191 PD patients

### 4.2. Analysis of 16S rRNA Sequence Data

The 16S rRNA gene was sequenced to enable bacterial identification. Adapter sequences were further removed using Trimmomatic v0.39 [31], and the reads were subsequently processed, aligned, and denoised using DADA2 v1.16 [32]. We filtered the reads with the DADA2 recommended parameters, then de-replicated and denoised using default settings. An amplicon sequence variant (ASV) table was generated, and taxonomic classification was conducted with the SILVA v132 database within DADA2. Species-level annotations were assigned using the addSpecies function with SILVA as the reference.

To ensure consistent sequencing depth across samples, rarefaction was performed at a threshold of 5000 reads per sample using the rarefy_even_depth function in phyloseq v1.32.0. Samples with fewer than 5000 reads were excluded before rarefaction. For downstream statistical analyses, sequence counts were normalized to relative abundance by dividing the number of sequences assigned to each ASV by the total sequence count in each sample. Only ASVs detected in at least 10% of samples were retained for further analysis.

### 4.3. Statistical Analysis

Demographic characteristics—including age, sex, antibiotic or probiotic use, and dietary intake (fruits, vegetables, grains, meats, nuts, and yogurt)—were compared between individuals with and without impulse behavior in the PD group. The Kruskal–Wallis test was used for continuous variables, while categorical variables were analyzed using the chi-square test. These analyses were performed using the tableone v0.13.2. To assess overall taxonomic diversity, alpha and beta diversity were calculated between the impulse and non-impulse groups. Alpha diversity metrics, including observed richness, Chao1, Shannon, and Simpson indices [33,34,35], were computed using phyloseq v1.50.0 [36] with *p*-values obtained through analysis of variance in stats v4.4.2. Beta diversity was evaluated using Canberra [37], unweighted UniFrac, weighted UniFrac [38], and Bray–Curtis distance [39], all computed with phyloseq v1.50.0 [36]. ADONIS analysis in the vegan v2.6.4 package was applied to determine statistical significance for beta diversity. Additionally, differences in microbial composition and relative abundance were analyzed within the PD group based on impulse status. A generalized linear model (GLM) with a negative binomial distribution and a zero-inflated model was used to identify significant variations in gut microbiota between impulse and non-impulse individuals, implemented using the glmmTMB v1.1.10. Network analysis was conducted to examine the associations between gut bacterial genera and impulsivity in PD patients. Based on the results from the differential abundance analysis (volcano plot), we selected genera that exhibited significant differences between the impulsive and non-impulsive groups for further exploration. Spearman’s rank correlation coefficients were calculated to assess pairwise relationships among genera. The resulting correlation matrix was visualized as a network graph, illustrating co-occurrence patterns among bacterial genera. Network analysis was conducted with igraph v2.1.4, tidygraph v1.3.1, and ggraph v2.2.1. Spearman and partial correlations were calculated using ppcor v1.1 to assess feature associations, and significant interactions were visualized as a network with ggraph v2.2.1.

### 4.4. Functional Enrichment Analysis of Predicted Metagenomes

Metagenome functional composition was predicted using Phylogenetic Investigation of Communities by Reconstruction of Unobserved States (PICRUSt2) v2.4.1 [40]. The standard pipeline was followed, which involved normalizing ASVs by copy number to account for variations in 16S rRNA gene copies among taxa, predicting functions based on Kyoto Encyclopedia of Genes and Genomes (KEGG) [41] orthologs, and categorizing predicted pathways according to KEGG hierarchical level 3. Within the PD group, metabolic pathway differences between individuals with and without impulse behavior were analyzed using Statistical Analysis of Metagenomic Profiles (STAMP) software v2.1.3 [42]. Comparisons were conducted using White’s non-parametric *t*-test (two-sided, 1000 replications), with statistical significance determined by a Storey false discovery rate (FDR) threshold of <0.05.

### 4.5. Data Availability and Ethical Statement

The sequencing data used in this study are publicly available in the European Nucleotide Archive (ENA) under accession number ERP016332. All data are fully anonymized and contained no identifiable personal information. In accordance with standard ethical guidelines for secondary analyses of de-identified public datasets, ethical approval was not required for this study.

## Figures and Tables

**Table 1:** Demographic characteristics and dietary habits of Parkinson’s disease patients with and without impulsive behaviors.

	Impulse (*n* = 14)	w/o Impulse (*n* = 177)	*p*-Value
**Gender**			0.089
Female	8 (57.1%)	55 (31.1%)	
Male	6 (42.9%)	122 (68.9%)	
**Age**			0.932
≤65	6 (42.9%)	67 (37.9%)	
>65	8 (57.1%)	110 (62.1%)	
**Antibiotics**			0.603
No	14 (100.0%)	165 (93.2%)	
Yes	0 (0.0%)	9 (5.1%)	
Missing	0 (0.0%)	3 (1.7%)	
**Probiotics**			0.111
No	8 (57.1%)	129 (72.9%)	
Yes	6 (42.9%)	36 (20.3%)	
Missing	0 (0.0%)	12 (6.8%)	
**Eat fruits or vegetable daily**			0.772
No	4 (28.6%)	37 (20.9%)	
Yes	10 (71.4%)	139 (78.5%)	
Missing	0 (0.0%)	1 (0.6%)	
**Eat grains daily**			0.501
No	6 (42.9%)	51 (28.8%)	
Yes	8 (57.1%)	123 (69.5%)	
Missing	0 (0.0%)	3 (1.7%)	
**Eat meats daily**			0.922
No	6 (42.9%)	76 (42.9%)	
Yes	8 (57.1%)	99 (55.9%)	
Missing	0 (0.0%)	2 (1.1%)	
**Eat nuts daily**			0.119
No	14 (100.0%)	135 (76.3%)	
Yes	0 (0.0%)	41 (23.2%)	
Missing	0 (0.0%)	1 (0.6%)	
**Eat yogurt daily**			0.685
No	12 (85.7%)	162 (91.5%)	
Yes	2 (14.3%)	14 (7.9%)	
Missing	0 (0.0%)	1 (0.6%)	
**Dopamine agonist**			0.171
No	10 (71.4%)	86 (48.6%)	
Yes	4 (28.6%)	91 (51.4%)	
**Carbidopa levodopa**			0.665
No	1 (7.1%%)	27 (15.3%)	
Yes	13 (92.9%)	150 (84.7%)	

## Data Availability

Data is contained within the article.
